# The Well-Born

**Published:** 1913-08

**Authors:** 


### The Well-Born.

So many people—people—in the world;
So few great souls, love ordered, well begun,
In answer to the fertile mother need!
So few who seem,
The image of the Maker's mortal dream;
So many born of mere propinquity—
Of lustful habit—or of accident.
Their mothers felt
No mighty, all-compelling wish to see
Their bosoms garden-places
Abloom with flower faces;
No tidal wave swept o'er them with its flood;
No thrill of flesh or heart; no leap of blood;
No glowing fire, flaming to white desire
For mating and for motherhood;
Yet, they bore children.
God! how mankind misuses thy command,
To populate the earth!
How low is brought high birth!
How low the woman; when, inert as spawn
Left on the sands to fertilize,
She is the means through which the race goes on!
Not so the first intent.
Birth, as the Supreme Mind conceived it, meant
The clear imperious call of mate to mate
And the clear answer. Only thus and then
Are fine, well-ordered, and potential lives
Brought into being. Not by church or State
Can birth be made legitimate,
Unless,
Love in its fullness bless.
Creation so ordains its lofty laws
That man, while greater in all things,
Is lesser in the generative cause.
The father may be merely man, the male;
Yet more than female must the mother be.
The woman who would fashion
Souls, for the use of earth and angels meet,
Must entertain a high and holy passion.
Not rank, or wealth, or influence of kings.
Can give a soul its dower
Of majesty and power,
Unless the mother brings,
Great love to that great hour.

—*Bulletin Texas State Board of Health.*

